# Direct measurement of absolute radiation pressure of leds in nanopascal range under ambient conditions with microcantilever

**DOI:** 10.1038/s41598-025-04812-9

**Published:** 2025-07-02

**Authors:** Yuki Takei, Hiromitsu Furukawa

**Affiliations:** National Institute of Advanced Industrial Science and Technology (AIST), 807-1, Shuku-machi, Tosu, 841-0052 Saga Japan

**Keywords:** Optical radiation pressure, Pressure sensor, Microelectromechanical systems (MEMS), Optical sensors, Optical manipulation and tweezers, Optical metrology

## Abstract

Optical radiation pressure caused by the force exerted by photons upon interacting with matter represents a crucial phenomenon for exploring physical and biological processes, particularly in the context of optical tweezers. However, accurately measuring the absolute value of radiation pressure presents a significant challenge owing to its inherently small magnitude. Conventional measurement setups are often complex and costly. This study demonstrates the measurement of radiation pressure using a near-infrared light-emitting diode (LED) in ambient conditions, employing a simple and cost-effective experimental setup. The radiation pressure was quantified by measuring the deflection of a highly sensitive microcantilever, achieving pressure measurements as low as 228 nPa, equivalent to a force of 21.5 fN. This represents the smallest absolute value of radiation pressure measured to date and marks the first successful application of an LED in such experiments under standard atmospheric conditions. The simplicity and affordability of this method make it a promising candidate for integration into compact devices for industrial applications, such as full-scale testing of sensitive pressure sensors used in atomic force microscopy, photoacoustic spectroscopy, and advancing the understanding of physical and biological phenomena.

## Introduction

Photons are massless but exert a discernible force known as radiation pressure^1–5^ when they interact with matter. This phenomenon is illustrated when a light of intensity 1 W/m² is directed perpendicularly at a surface and fully reflected, generating a pressure of 6.7 nPa, as described by the equation:

1 $$\:P=\:\frac{2{I}_{\text{L}}}{c},$$

where *P* represents the radiation pressure, *I*_L_ is the light intensity, and *c* is the speed of light in a vacuum. Generally, the radiation pressure depends on the optical properties of the surface: reflected photons impart twice as much momentum as absorbed photons, while transmitted photons impart no momentum change, as quantitatively analysed and experimentally verified in previous studies^6,7^. Though this force is minute and undetectable by touch, its effects are visually evident, such as in the tail formation of comets. The precise, contact-free application of radiation pressure has been utilized in a variety of technologies, including optical tweezers^8–12^laser cooling^13–16^and solar sails^17–20^. Specifically, optical tweezers are crucial for particle alignment^21,22^ and for force measurements in biological studies^23–27^as well as in the exploration of diverse physical interactions^28,29^.

Various innovative methods have been proposed for measuring radiation pressure^30–38^. Techniques such as interferometry have been employed to quantify the displacement of latex beads within light-encapsulated droplets^30^while enhancements to atomic force microscopy (AFM) incorporate lasers to induce deflections in microcantilever^31,37,38^. Other methods have used macroscopic mechanical oscillators and torsion balances^33^. In high vacuum environments, forces as low as sub-femtonewtons have been detected using lasers^36^. Although these methods represent significant advancements, accurately measuring such low levels of radiation pressure remains a formidable challenge, often necessitating complex measurement systems.

Conventionally, measurements of radiation pressure have employed lasers as the primary light sources. To date, the use of LEDs for this purpose has not been documented. Although LEDs have lower spatial coherence than lasers, making it difficult to focus their light and observe radiation pressure, they offer several advantages. LEDs can be directly modulated by adjusting the input current, eliminating the need for additional components like optical choppers. This feature provides flexibility in generating pressure patterns. Furthermore, LEDs present a cost-effective alternative to laser-based systems, which could prove invaluable in industrial applications requiring large-scale implementation. The affordability of LED setups allows for comprehensive testing of numerous pressure sensors or microcantilevers, enabling widespread application rather than limited sampling-based approaches. This approach contrasts sharply with the use of expensive equipment such as nanoindenters and offers the significant advantage of enabling measurements under ambient conditions. Many conventional methods necessitate precisely controlled environments to avoid interference, which adds complexity and limits their practical applicability. Such advancements can have significant industrial applications, notably in the high sensitivity quality assurance of microcantilevers used in AFM or in pressure sensors deployed in photoacoustic spectroscopy through rigorous testing.

**Fig. 1:**
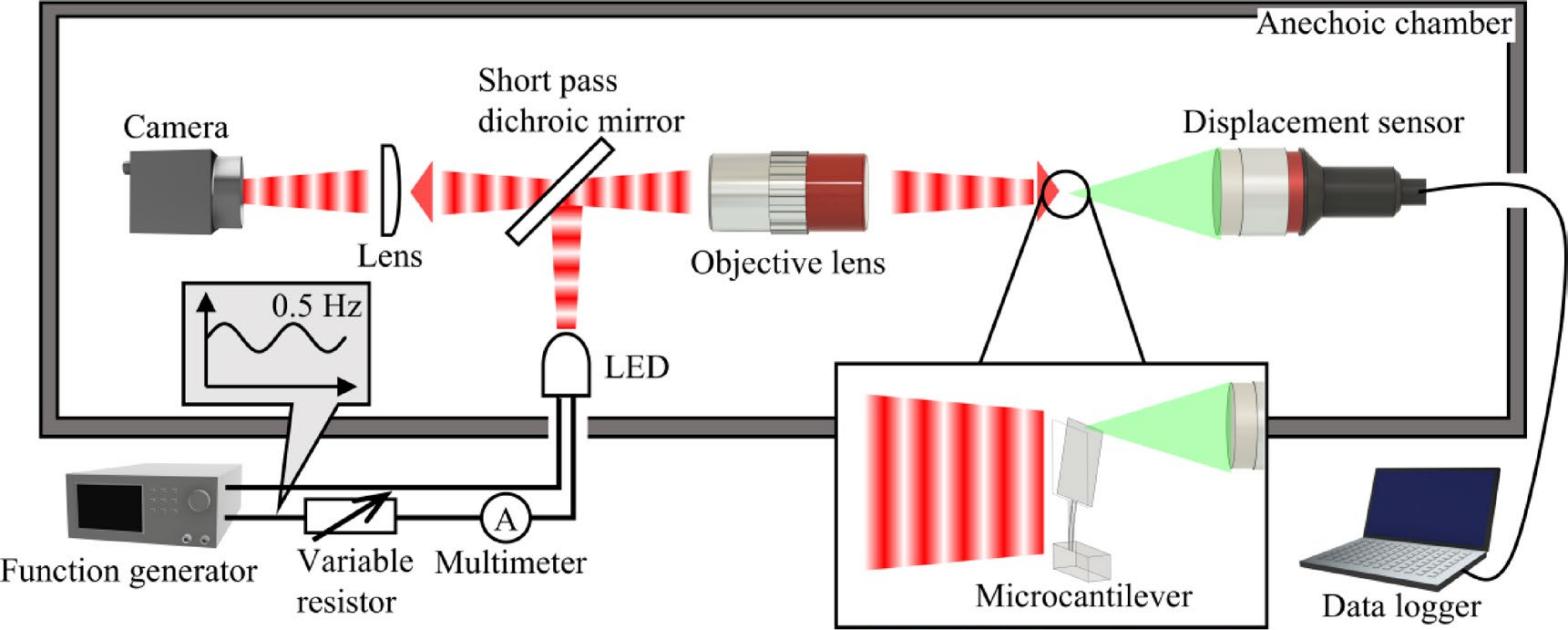
Schematic of the measurement system. This diagram illustrates the use of a microcantilever as a transducer for measuring optical radiation pressure, with all measurements conducted under ambient conditions.

This study introduces and experimentally validates a method to measure the radiation pressure produced by an LED. This was achieved under ambient conditions using a streamlined setup that incorporates an exceedingly sensitive microcantilever. To maximise sensitivity to extremely small pressures, the microcantilever was designed to be long and thin, with a wider area near its free end to enhance deflection response under applied pressure. It had a total length of 500 μm, thickness of 0.34 μm, and estimated spring constant of 0.26 mN/m. Combined with the experimental apparatus, this enabled pressure measurement as low as hundreds of nPa. Details of the design parameters are provided in the Methods section. Figure 1 depicts the schematic of the experimental apparatus. The setup includes a near-infrared LED (AMS OSRAM, SFH 4550), which projects light onto one side of a silicon wafer-crafted microcantilever. The resulting deflection, induced by the radiation pressure, is measured from the opposite side using a laser displacement sensor (KEYENCE, CL-P015), with a nominal resolution of 1 nm and maximum sampling rate of 10 kHz. By employing frequency modulation and Fourier analysis, sub-nanometre deflections corresponding to the radiation pressure were reliably quantified. Notably, our configuration did not necessitate a vacuum chamber, and all experiments were conducted under standard atmospheric conditions. The selected LED, emitting at a peak wavelength of 860 nm, minimizes light absorption by the microcantilever. It features a narrow emission half-angle of ± 3° and a maximum radiant flux of 70 mW. The LED operation was controlled by a function generator (SIGLENT, SDG 1032X), with current measurements taken by a multimeter (SIGLENT, SDM 3055). To reduce noise interference from sound and air currents, the bulk of the setup was constructed within an anechoic chamber, despite the wiring connections to the LED and the displacement sensor extending outside. All components within the chamber were installed on an anti-vibration table to further minimize disturbances. Light from the LED was reflected off a short-pass dichroic mirror (Thorlabs, DMSP650) and channeled through an objective lens (Mitutoyo, M Plan Apo NIR 10X). The short-pass dichroic mirror in the setup primarily reflected the near-infrared emission from the LED. However, the cantilever could still be visualised without auxiliary illumination, owing to residual short-wavelength components reaching the camera. This setup allowed the collimated light beam from the lens to be focused onto the microcantilever. During experiments, the spot size was adjusted using a camera to ensure it exceeded the dimensions of the microcantilever, thereby uniformly illuminating it. The position of the displacement sensor was strategically placed at the microcantilever’s tip to capture maximum deflection.

## Results

**Fig. 2:**
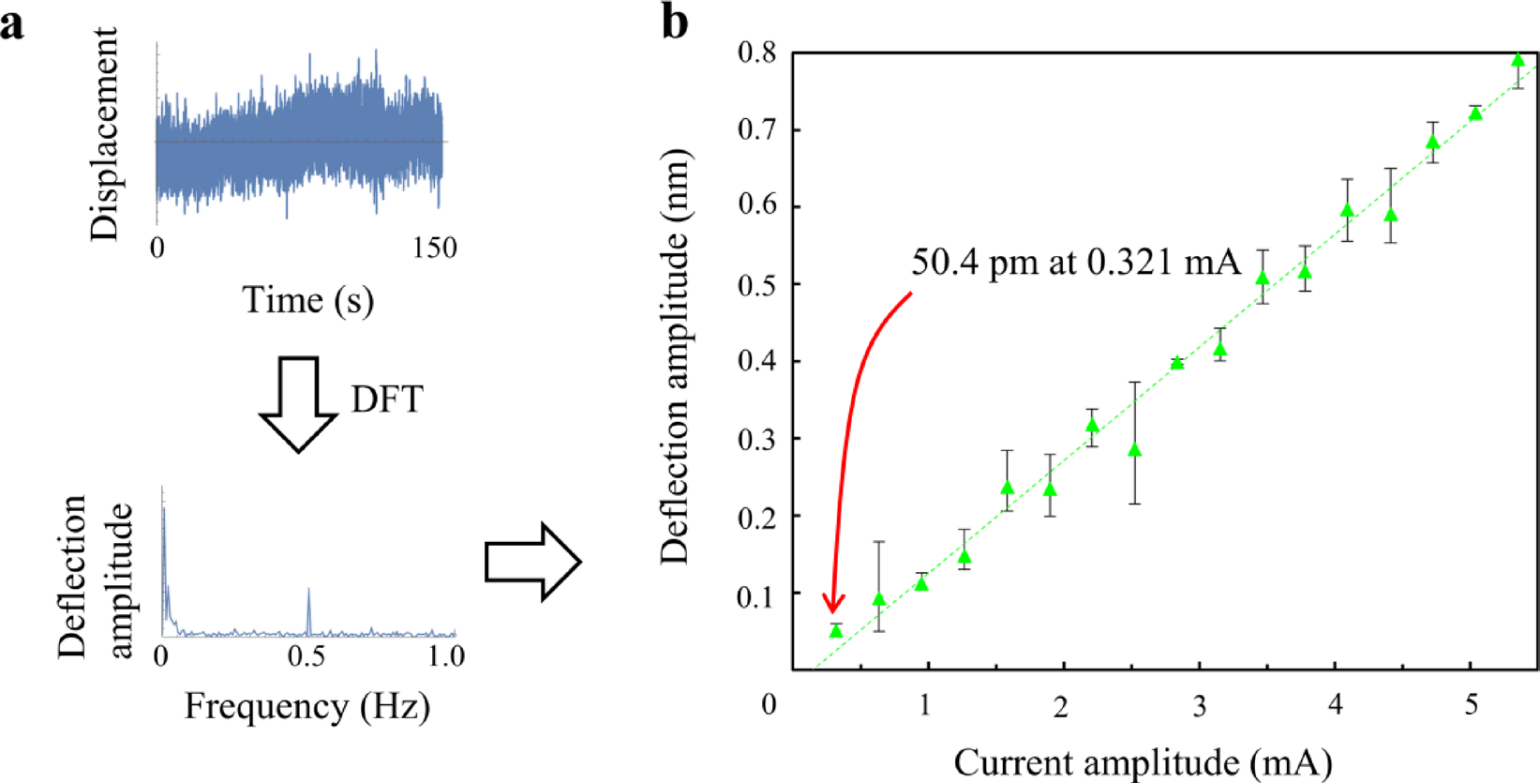
Microcantilever deflection induced by radiation pressure. (**a**) Process of the deflection analysis. Measured displacements were median filtered to reduce noise before applying the discrete Fourier transform (DFT). (**b**) Analysis of deflection amplitude as a function of current amplitude. This plot aggregates the average results from three DFT analyses of the deflection measurements, highlighting the smallest recorded deflection of 50.4 pm at a current amplitude of 0.321 mA.

Subsequent experiments aimed to determine the measurement capability of the apparatus for measuring radiation pressure. To reduce the impact of ambient noise, the voltage supplied to the LED was modulated in a sinusoidal waveform. Displacement measurements were then recorded over an extended duration of 150 s and analyzed using Fourier analysis. Considering the microcantilever’s light weight relative to its surface area, it was anticipated that air resistance would dampen high-speed vibrations. Therefore, the modulation frequency was carefully set to a low 0.5 Hz based on finite element method (FEM) analysis considering air damping (see Methods). This frequency is sufficiently below the microcantilever’s resonant frequency, strategically avoiding both the resonant frequency and pervasive low-frequency environmental noise. The nominal rise and fall times of the LED were 12 ns, adequately brief for our purposes. The sinusoidal bias voltage was maintained at 3.0 V, with amplitude adjustments ranging from 50 to 850 mV in increments of 50 mV. Each experimental condition was replicated three times to ensure reliability. The displacement sensor operated at a sampling rate of 10 kHz. Noise interference was curtailed using a median filter with a window size of 256, producing a dataset of 1.5 million points for deflection measurements. Discrete Fourier transform (DFT) was applied to these data, and the deflection amplitude was calculated by normalizing the 0.5 Hz component of the results, as shown in Fig. 2 (a). Figure 2 (b) illustrates the correlation between the current amplitudes and the microcantilever deflection amplitudes. In this figure, triangular markers denote the average deflection amplitude from three measurements, while the dashed line indicates the linear regression analysis. Vertical lines on each marker point out the variation observed under each experimental condition. Preliminary tests confirmed the proportionality between current and radiant flux, supporting the hypothesis that radiant flux correlates with radiation pressure. This observation is corroborated by the proportionality between the deflection amplitude and current amplitude demonstrated in Fig. 2 (b), aligning with theoretical predictions. Moreover, the regression line’s proximity to the origin supports the validity of Eq. (1).

The minimal deflection amplitude, as shown in Fig. 2 (b), was 50.4 pm at a current amplitude of 0.321 mA, corresponding to a pressure amplitude of 228 nPa across the entire microcantilever. The pressure *P* and the displacement *y* are related by *P* = *y*/(220.5 × 10^−6^), as detailed in the Methods section. The force exerted by this pressure, calculated by integrating over the microcantilever’s surface area, was found to be 21.5 fN. With the LED emitting a radiant flux of 16.3 µW at 0.321 mA, theoretical calculations suggest that the microcantilever should ideally experience a pressure of 1114 nPa, or a force of 107.4 fN, under conditions where the light is completely incident upon and perfectly reflected by the microcantilever. Therefore, the observed radiation pressure is approximately 20% of the theoretical value for perfect reflection. This value can be attributed to the spot size of the LED and the optical properties of the microcantilever. Assuming the spot size of the LED just covers the microcantilever, approximately 35.2% of the total radiant flux is expected to impinge on its surface. Furthermore, considering the Fabry–Pérot cavity effect in the thin silicon structure, where multiple internal reflections modify the effective reflectance and absorbance, calculations based on the Fresnel equations at a wavelength of 860 nm estimate that 27.8% of the incident photons are reflected and 2.75% are absorbed by the cantilever^39,40^. Consequently, the radiation pressure on the cantilever is predicted to be approximately 58.3% of that under perfect reflection, resulting in an overall value of approximately 21% when combined with the geometric overlap factor. This estimation closely matches the experimental result, thereby supporting the validity of the optical power transfer model employed in this study. As shown in Fig. 2 (b), a linear regression analysis was conducted to determine the relationship between measured deflection amplitudes and applied current amplitudes. The coefficient of determination (R² value) was 0.99, demonstrating that 99% of the variability in the dataset can be explained by this model, which further supports the reliability of the experimental setup and measurements. Throughout the experiment, the DC bias current through the LED was approximately 10.79 mA. Assuming that 20% of the ideal pressure was achieved under the experimental conditions, the microcantilever consistently registered an absolute pressure of 7135 nPa or a force of 671 fN. However, it should be noted that the DC bias can be reduced as the deflection caused by the DC bias current is not factored into the DFT; only the amplitude is influenced. Therefore, it is possible to measure absolute pressures and forces corresponding to modulation amplitudes of 228 nPa or 21.5 fN.

The variability in the measurements presented in Fig. 2 (b) did not show an increasing trend with smaller displacements or reveal any distinct patterns across the dataset, suggesting that the observed variability primarily stems from random noise. Therefore, while the simplicity of the measurement technique is advantageous, enhancing the number of observations, extending the duration of measurements, or optimizing for a lower noise environment can facilitate the detection of even smaller radiation pressures.

The microcantilever can theoretically respond to optical modulation at kilohertz frequencies. However, unlike typical long and narrow cantilevers, it is more susceptible to viscous air damping under ambient conditions, where the damping force increases with vibration velocity. As detailed in the Methods section, this is attributed to the broader surface area near the free end of the cantilever, which significantly reduces oscillation amplitudes at higher modulation frequencies, making reliable detection difficult. Moreover, operation near resonance can amplify dynamic effects and compromise measurement stability. Therefore, low-frequency operation was selected to achieve stable and highly sensitive radiation pressure measurements.

However, because the magnitude of radiation pressure is independent of modulation frequency, these measurements can then be extrapolated to apply to light modulated at higher frequencies to ascertain the radiation pressure, as validated by the experiments detailed earlier. Therefore, the apparatus can produce pressure through both frequency and intensity modulation by controlling the input current. This flexibility allows to create complex pressure patterns, such as high-frequency modulation superimposed on a low-frequency carrier wave, enabling more intricate and dynamic radiation pressure profiles. Although systematic experimental evaluation of potential frequency-dependent effects, including damping, remains a subject for future work, the ability to precisely quantify quasi-static radiation pressure provides a strong foundation for these advanced applications.

The optical properties of the light source are also critical factors influencing the precision and limits of radiation pressure measurements. Owing to their high spatial coherence and narrow spectral bandwidth, lasers enable more precise focusing and consistent optical interactions with the cantilever, making them theoretically more advantageous for high-precision measurements. However, this study demonstrates that despite the broader emission profile and spectral bandwidth inherent to LEDs, precise and reproducible measurements of LED-induced radiation pressure can be successfully achieved under ambient conditions. This finding highlights the potential of LED-based systems as reliable and cost-effective radiation pressure sources for future precision applications, while suggesting that further improvements in source characteristics could enhance measurement capabilities in even more demanding scenarios.

## Conclusion

This study experimentally demonstrated a novel approach for measuring the radiation pressure exerted by LEDs. The ability of the approach to measure pressures as low as 228 nPa using a simplified setup not only simplifies the experimental process but also reduces the associated costs, presenting a significant advantage over conventional laser-based systems. Crucially, this system is designed to measure pressure as an absolute value rather than merely a resolution limit, which is significant for practical applications. The potential applications of this method are extensive, ranging from full-scale testing of pressure sensors to enhancing our understanding of various physical and biological phenomena. While this study demonstrated the system’s versatility through successful testing under ambient conditions, further enhancements are necessary to fully realize its capabilities. Future work will focus on reducing noise and optimizing the microcantilever structure to expand the detection limits of this promising technology.

## Methods

**Fig. 3:**
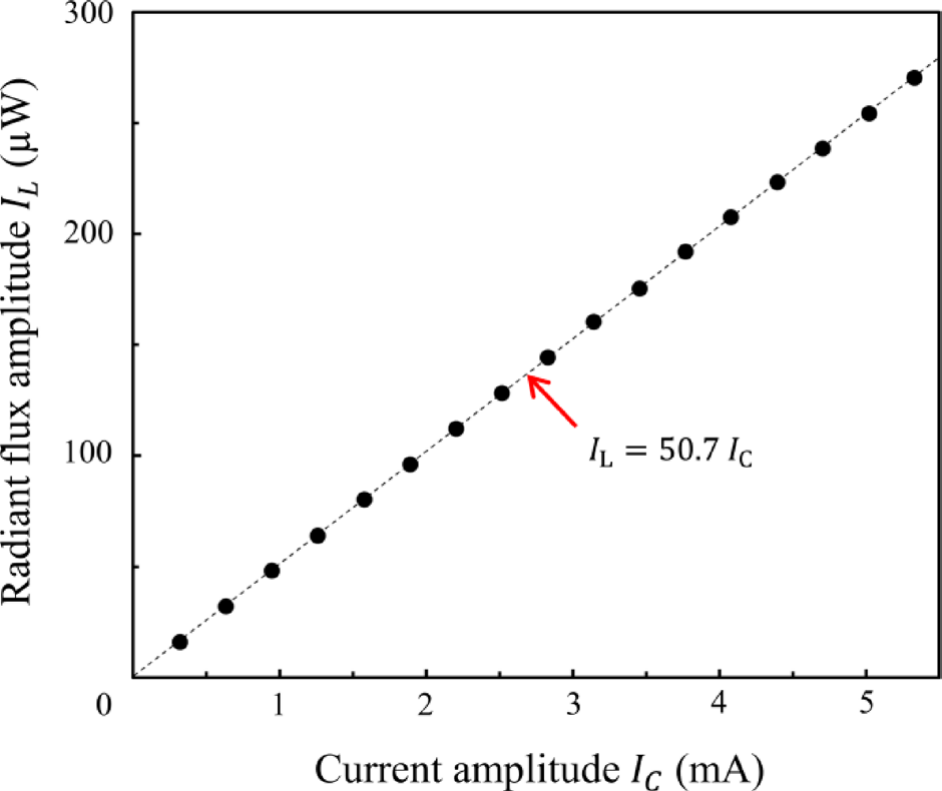
Relationship between radiant flux and current supplied to the LED. The radiant flux of 16.3 µW at a current amplitude of 0.321 mA is determined from the regression line.

The light emitted by the LED attenuates along the optical path before reaching the microcantilever. Consequently, the relationship between the radiant flux and the current supplied to the LED was quantified prior to experimentation by positioning a photodiode (Ophir, PD300-BB) at the microcantilever’s location, as depicted in Fig. 1. The modulation frequency was set at 0.5 Hz, consistent with the experimental parameters. The correlation between the current amplitudes *I*_C_ and the radiant flux amplitudes *I*_L_ is shown in Fig. 3. At a current of 0.321 mA, the microcantilever was subjected to a radiant flux of 16.3 µW.

When a uniformly distributed load *w* is applied to a cantilever beam with uniform width *b*, thickness *h*, and length *l*, the deflection *y* at the free end can be calculated using the following equation^41^:

2 $$\:y=\frac{3}{2}\:\frac{w\:{l}^{4}}{E\:b\:{h}^{3}},$$.

where, *E* represents the Young’s modulus of the cantilever material. The distributed load *w* represents the force per unit length and can be expressed as *P* × *b*, where *P* is the applied pressure. The design of the microcantilever used in this experiment was based on this principle. It suggests that a thin, long cantilever with a large surface area will exhibit greater deflection under pressure. However, such a design increases the impact of air resistance, limiting the cantilever’s ability to vibrate at high frequencies under standard atmospheric conditions.

**Fig. 4:**
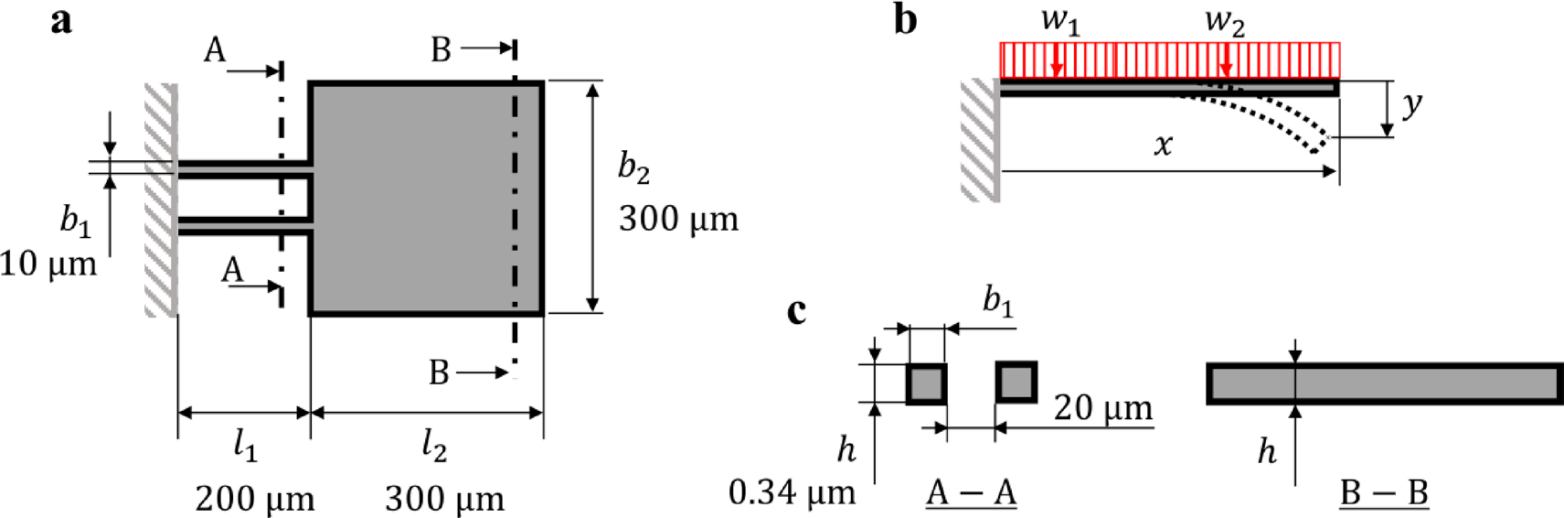
Design parameters of the microcantilever. (**a**) Top view showing the base of the microcantilever is shaded to highlight the primary support area. (**b**) Cross-sectional view detailing the distributed loads *w*_1_ and *w*_2_ exerted by radiation pressure. (**c**) Labeled cross-sectional view corresponding to the top view, detailing structural dimensions.

Figure 4 outlines the design parameters of the microcantilever, which consists of two connected cantilever segments, each with differing dimensions. The first segment has a width of 2*b*_1_ and a length *l*_1_, while the second segment has a width of *b*_2_ and a length *l*_2_. Owing to the varying areas of these segments, a uniform pressure applied to one side of the microcantilever results in different loads across the two sections. The deflection at the tip of the microcantilever (at *x* = 500 μm) under pressure *P* can be calculated by superposing the deflections of the two cantilever segments by extending Eq. (2) to

3 $$\begin{aligned}\:y=\left(\frac{1}{4E\:{b}_{1}\:{h}^{3}}\right)& \Big({w}_{1}\:{x}^{4}-4{w}_{1}\:{x}^{3}\left({l}_{1}+{l}_{2}\right)+6{w}_{1}\:{x}^{2}{\left({l}_{1}+{l}_{2}\right)}^{2}\\ &-12{l}_{1}\:{l}_{2}\:x\left({l}_{1}+{l}_{2}\right)\left({w}_{1}-{w}_{2}\right)\\ &+2{{l}_{1}}^{2}\:{l}_{2}\:\left(2{l}_{1}+3{l}_{2}\right)\left({w}_{1}-{w}_{2}\right)\Big),\end{aligned}$$

where *E* represents the Young’s modulus of the cantilever material (Si: 131 GPa), and the distributed loads *w*_1_ and *w*_2_ are derived from the pressure *P* as 2*b*_1_ *P* and *b*_2_ *P*, respectively. This equation reduces to Eq. (2) under the conditions *l*_1_ + *l*_2_ = *l* = *x* and *w*_1_ = *w*_2_ = *w*. Substituting the design parameters into Eq. (3) allows for the derivation of the following relationship:

4 $$\:P=\frac{y}{220.5\times\:{10}^{-6}}.$$

Therefore, given the measured deflection, the pressure can be calculated from Eq. (4). For example, with a deflection of 50.4 pm, the pressure was determined to be 228 nPa. The sensitivity of this measurement was calculated as 0.221 pm/nPa based on this relationship.

**Fig. 5:**
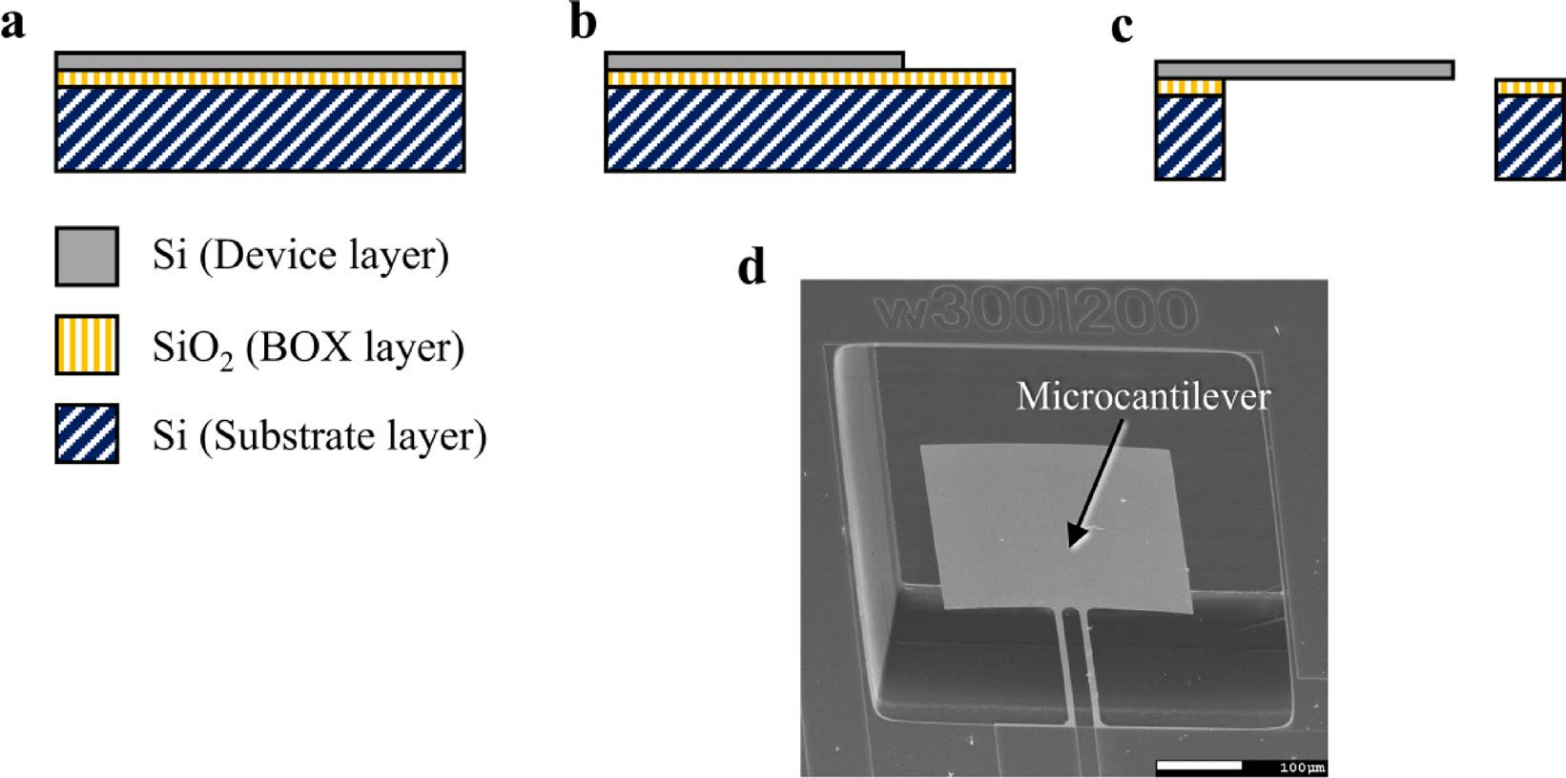
Overview of the microcantilever fabrication processes and the final product. (**a**) The microcantilever was constructed from a silicon-on-insulator wafer. (**b**) The outline of the microcantilever was patterned on the device layer. (**c**) Fabrication involved the removal of the substrate layer and the buried oxide (BOX) layer. (**d**) Scanning electron microscopy image of the completed microcantilever.

The microcantilever was fabricated using standard silicon-on-insulator (SOI) microelectromechanical system (MEMS) techniques, as detailed in Fig. 5. It was fabricated from an SOI wafer, featuring a device layer that is 0.34 μm thick (Fig. 5(a)). During its manufacture, the device layer underwent deep reactive ion etching (DRIE), as illustrated in Fig. 5(b). This was followed by DRIE processing of the substrate layer, with the microcantilever subsequently released by removing the buried oxide (BOX) layer using vaporized hydrofluoric acid (Fig. 5(c)). Consequently, the final structure of the microcantilever comprised a single layer of silicon with a thickness of 0.34 μm. A scanning electron microscopy image of the completed microcantilever is presented in Fig. 5(d).

**Fig. 6:**
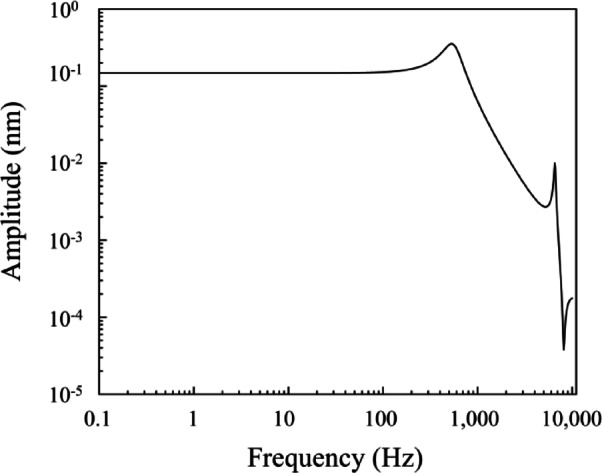
Simulated frequency response of the microcantilever under a uniform pressure load of 1 µPa, considering air damping. The results indicate that resonance-induced amplification effects are negligible below 40 Hz, validating the selection of 0.5 Hz as the modulation frequency.

The modulation frequency of 0.5 Hz was selected based on the results of a FEM simulation (ANSYS Inc., ANSYS) that incorporates mass-proportional damping (α damping), which approximates linear viscous behaviour at low frequencies. As shown in Fig. 6, no significant amplitude enhancement due to resonance occurred below approximately 40 Hz. Therefore, operating at 0.5 Hz effectively avoids dynamic amplification of the cantilever’s deflection.

## Data Availability

The datasets generated during and/or analyzed during the current study are available from the corresponding author on reasonable request.
